# The Neuropeptide-Related HERC5/TAC1 Interactions May Be Associated with the Dysregulation of lncRNA GAS5 Expression in Gestational Diabetes Mellitus Exosomes

**DOI:** 10.1155/2022/8075285

**Published:** 2022-02-08

**Authors:** Gui-Yan Tang, Ping Yu, Chen Zhang, Hong-Yu Deng, Mei-Xiu Lu, Jiang-Hua Le

**Affiliations:** ^1^Department of Obstetrics, Affiliated Hospital of Guilin Medical University, China; ^2^Department of Pelvic Floor Rehabilitation, The Second Affiliated Hospital of Guilin Medical University, China; ^3^Department of Endocrinology, Affiliated Hospital of Guilin Medical University, China; ^4^Department of Obstetrics, The Second Affiliated Hospital of Guilin Medical University, China

## Abstract

**Objective:**

The goal of this work was to look at the expression and probable role of exosomal long noncoding RNA (lncRNA) GAS5 in gestational diabetes mellitus (GDM), as well as forecast the importance of its interaction with neuropeptides in the progression of the disease.

**Methods:**

We divided 44 pregnant women visiting the obstetric outpatient clinics at the Affiliated Hospital of Guilin Medical College from January 2021 to December 2021 into healthy and GDM groups. We measured the expression levels of the lncRNA GAS5 in peripheral blood using PCR and compared the expression levels between the 2 groups. The Gene Expression Omnibus (GEO) database and the R software were used to analyse the differences in the genes expressed in the amniotic fluid cells in the GDM and normal groups. catRAPID was used to identify potential target proteins for GAS5. Key neuropeptide-related proteins and potential target proteins of GAS5 were extracted, and protein interaction networks were mapped. AlphaFold 2 was used to predict the structure of the target protein. The ClusPro tool was used to predict protein-protein interactions. ZDOCK was used to further confirm the protein–nucleic acid docking.

**Results:**

The lncRNA GAS5 was downregulated in the peripheral blood of pregnant women with GDM compared with normal pregnant women. The subcellular localization sites of GAS5 were the nucleus, cytoplasm, and ribosome; in addition, GAS5 was present in exosomes. Intercellular interactions, including neuropeptide receptors, were increased in the amniotic fluid cells of patients with GDM. Venn diagram analysis yielded seven neuropeptide-related proteins and three GAS5 target proteins. Among them, HERC5/TAC1 interacted and GAS5 docked well with HERC5.

**Conclusion:**

The lncRNA GAS5 in the peripheral blood exosomes in patients with GDM may be a new target for the detection of GDM, and the interaction between GAS5 and HERC5/TAC1 may be involved in the pathogenesis of GDM.

## 1. Introduction

Gestational diabetes mellitus (GDM) is a common complication that occurs in pregnant women in their middle and late stages of pregnancy and is mainly caused by disorders of glucose metabolism in the body [1]. Previous studies have shown that GDM has a variety of adverse maternal and infant outcomes such as giant babies, stillbirths, excessive amniotic fluid, hypertensive disorders of pregnancy, postpartum haemorrhage, and an increased risk of future metabolic syndrome and diabetes for both the patient and her foetus [2–5]. Insulin resistance (IR) is an important pathological mechanism involved in the development of GDM [6, 7]. However, the etiopathogenesis of GDM has not been clarified, and accurate and sensitive early diagnosis methods and effective disease monitoring indicators have not been established thus far.

The role and mechanism of exosomes in the pathogenesis of GDM is one of the important topics being investigated in recent studies. Compared with normal pregnant women, those with GDM showed increased levels of placenta-derived exosomes. High glucose levels, hypoxia, and obesity are involved in inducing an increase in exosomes during pregnancy, and exosomes may be used as biomarkers for the diagnosis and treatment of gestational diabetes [8, 9]. In addition to the differences in the exosome levels during pregnancy, significant differences were also observed in exosome contents [10]. In particular, exosomes secreted from the placenta contain Fas and TRAIL molecules, which induce apoptosis of Jurkat T cells and monocytes in a dose-dependent manner and regulate the immune status of the body [11]. In addition, trophoblast-derived exosomes can promote vascular endothelial cell migration and lumen formation [10]. Therefore, the results of previous studies indicate that exocrine complex-containing substances can be involved in the mechanistic regulation of maternal pregnancy through multiple pathways. We speculated that exosomes or specific active substances contained within exosomes may be a new form of pathological manifestation of GDM. To date, however, the composition of active substances present in the plasma exosomes during pregnancy in GDM patients is not completely understood, and further studies are required to understand the relationship between the substances present in exosomes and the pathological manifestations of GDM.

Long noncoding RNA (lncRNA) is an important endosomal component in exosomes [12, 13]. The lncRNA GAS5 is approximately 651 nt long and is an important regulator of cell proliferation and growth. A recent study showed that GAS5 levels were downregulated in the serum of patients with type 2 diabetes mellitus (T2DM) [14]. GAS5 expression is significantly reduced in the peripheral blood and renal tissues of patients with DM and diabetic nephropathy. Reduced GAS5 expression, hypertrophy of mesangial cells, and increased matrix synthesis are observed in glomerular mesangial cells cultured in vitro with high glucose treatment [15, 16]. Therefore, GAS5 plays an important role in diabetes and diabetic vascular complications and may be a new diagnostic target for diabetes. However, the relevance of GAS5 in the development of GDM remains to be clarified.

Recent studies showed that neuropeptide levels are closely related to GDM. It has been shown that neuropeptide Y (NPY) plays an important role in the development of DM [17]. NPY is a very conserved neuropeptide and an important neurotransmitter and a proappetite factor, which can cause obesity by increasing food intake. Obesity is one of the important risk factors for the development of GDM [18, 19]. In addition, the levels of galanin, a neuropeptide, are significantly increased in the plasma of pregnant women with GDM and are positively correlated with fasting glucose levels [20]. The neuropeptide cortistatin has an inhibitory effect on insulin secretion [21, 22]. Cortistatin levels are decreased in GDM, and it plays a potential role in the pathogenesis of GDM [23]. These findings suggest that neuropeptides are involved in the pathogenesis of GDM. lncRNAs regulate protein expression; however, the function of GAS5 in protein binding has not been clarified thus far [24–26]. Based on the differences in the exosome levels in pregnant women with GDM and the role of GAS5 in diabetes, we hypothesised that the molecular interaction of exosomes in pregnant women with GDM may mediate the development of GDM through the delivery of GAS5-regulated neuropeptide-related proteins.

In this study, we investigated in detail the relevance of exosomal lncRNA GAS5 in the development of clinical GDM in pregnant women and the potential functions of neuropeptides in GDM using clinical studies and bioinformatics. Our results facilitate the understanding of the pathogenesis of GDM and provide new ideas for the clinical diagnosis and treatment of the disease.

## 2. Materials and Methods

### 2.1. Clinical Study Subjects

We included pregnant women visiting the obstetric outpatient clinics at the Affiliated Hospital of Guilin Medical College from January 2021 to December 2021 in this study. The women were divided into healthy pregnant women and GDM groups. Among them, 23 were healthy pregnant women, aged 30–38 years, and 21 were pregnant women with GDM, aged 30–40 years. The study was approved by the Ethics Committee of the Second Affiliated Hospital of Guilin Medical College, and a signed informed consent form was obtained from all participants. Diagnostic criteria for GDM included no history of diabetes before pregnancy, 75 g glucose tolerance test at 24–28 weeks of pregnancy, and fasting blood glucose ≥ 5.1 mmol/l (1 h OGTT ≥ 10.0 mmol/l and 2 h OGTT ≥ 8.5 mmol/l); blood glucose levels at any aforementioned time point were considered a criterion for GDM diagnosis. Age, infertility duration, BMI (kg/m [2]), peripheral blood levels of GAS5, LH (mIU/ml), FSH (mIU/ml), LH/FSH, E2 (pg/ml), P (pg/ml), FPG (mg/dl), 2h OGTT (mg/dl), HbA1c (%), IL-6 (pg/ml), TNF-*α* (pg/ml), and CRP (mg/l) were collected from the enrolled pregnant women.

### 2.2. Inclusion and Exclusion Criteria

Inclusion criteria included the following: (1) gestational duration ≤ 28 weeks, (2) pregnant women aged 22–45 years, (3) providing samples for complete blood count and visiting our hospital for regular maternity checkups and delivery, (4) pregnant women who were able to complete the 75 g oral glucose tolerance test (OGTT), (5) pregnant women with a diagnosis of GDM who had good glycaemic control through diet or exercise, and (6) those who signed an informed consent form.

Exclusion criteria included the following: (1) multiple pregnancies on ultrasonography; (2) pregnant women with missing case information; (3) history of other metabolic disorders such as hyperthyroidism, haematologic disorders, and liver disease; (4) acute infection; and (5) stress injury or use of special medications.

### 2.3. Dataset Acquisition and Variance Analysis

The Gene Expression Omnibus (GEO) database was used to analyse changes in gene expression in women with GDM. The GSE150621 is a dataset targeting specific RNA sequencing in amniotic fluid cells in the GDM and normal groups. Variance analysis was performed using the “DESeq2” package of R software [27]. Genes meeting the threshold of ∣log_2_(FC) | >1 and *p* < 0.05 were defined as differential genes. Neuropeptide-related genes were identified using the GeneCards database. A search using the keyword “neuropeptide” yielded 3165 neuropeptide-related genes [28]. Finally, features scoring >5 for neuropeptide-related genes were extracted.

### 2.4. Subcellular Localization of GAS5

LncSEA includes over 40,000 lncRNA datasets from 18 different categories (miRNA, drug, disease, methylation patterns, cancer-specific phenotypes, lncRNA-binding proteins, cancer markers, subcellular localization, etc.). This dataset was used to analyse the subcellular localization of GAS5 [29].

### 2.5. Enrichment Analysis

Gene Ontology (GO) and Kyoto Encyclopedia of Genes and Genomes (KEGG) were used for enrichment analysis using the clusterProfiler package of the R software (version 3.14.3); species were used with *Homo sapiens*, and *p* < 0.05 was defined as highly enriched pathways [30]. HALLMARK, KEGG, and GO gene sets for enrichment analysis were obtained using the gene set enrichment analysis (GSEA) from the Broad Institute website. GSEA software (V4.0.2) was used to analyse the gene set enrichment of amniotic fluid cells in patients with and without diabetes. Samples with *p* < 0.05 and false discovery rate (FDR) *q* < 0.05 were considered statistically significant [31].

### 2.6. Extraction and Detection of GAS5 from Peripheral Blood

The lncRNA GAS5 was extracted from peripheral blood using cellular Trizol RNA extraction reagent. The expression levels of GAS5 were quantified using reverse transcription polymerase chain reaction (RT-PCR) according to the manufacturer's instructions. The relative expression of miRNAs was calculated using the 2^-*ΔΔ*Ct^ method.

### 2.7. Prediction of the Structure of Proteins and Nucleic Acids

In the critical assessment of structure prediction of most proteins using AlphaFold 2, CASP 14 differed from the real structure by a width of only one atom and reached a level predicted by human observation using sophisticated instruments such as cryoelectron microscopy [32]. To date, the structures of more than 98.5% of human proteins have been predicted using AlphaFold 2. In this study, we used the AlphaFold 2 dataset to predict the protein structures of the target genes. The nucleic acid sequence of lncRNA GAS5 was identified using the NCBI database [33]. RNAalifold was used to complete the secondary structure prediction of lncRNA GAS5 [34]. We used 3 automated methods developed by Xiao Lab, namely, 3dRNA, 3dRPC, and ASPDock, to predict the 3D structures of the noncoding RNA, RNA–protein complex, and protein–protein complexes, respectively, and these methods were used to predict the tertiary structure of the lncRNA GAS5 [35]. The structure files of all nucleic acid proteins were saved in protein data bank (PDB) format.

### 2.8. Prediction of Interactions between Biological Macromolecules

Protein–protein interaction networks were mapped using the STRING database [36] and predicted using ClusPro tools [37]. catRAPID is an algorithm for estimating the propensity for protein–RNA binding. By combining secondary structure, hydrogen bonding, and van der Waals forces, the protein–RNA binding can be predicted using this algorithm with high accuracy [38]. This database was used to identify potential target proteins for GAS5. ZDOCK (version 3.0.3) determines all translational and rotational spaces between the 2 biomolecules and then assigns a score for each possible pose. The scores are calculated using an energy-based scoring function that calculates the potential energy, spatial complementarity, and electric field forces [39]. ZDOCK was used to further confirm the protein–nucleic acid docking. Docking results between biomolecules were exported as PDB files and were visualised using PyMOL [40].

### 2.9. Statistical Analysis

Statistical analysis was performed using the R software version 3.6.0. Quantitative data were expressed as mean ± standard deviation if they were normally distributed, and an independent samples *t*-test was used to compare the means between the two groups. Spearman correlation analysis was performed, and a positive *r* value indicated a positive correlation, whereas a negative *r* value indicated a negative correlation. Qualitative data were expressed as frequencies, and the chi-square test was used to compare two independent groups of dichotomous variables. As described in previous studies, we used support vector machines (SVMs) to identify key biomarkers using the R software packages “e1071,” “kernlab,” and “caret” in “svmRadial” [41–43]. The receiver operating characteristic (ROC) curve was used to test the early predictive efficacy of the target factor for GDM. A *p* value < 0.05 was considered statistically significant.

## 3. Results

### 3.1. Downregulation of GAS5 Expression in GDM Patients and Enrichment Characteristics of Amniotic Fluid Cells in GDM

**Figure 1:**
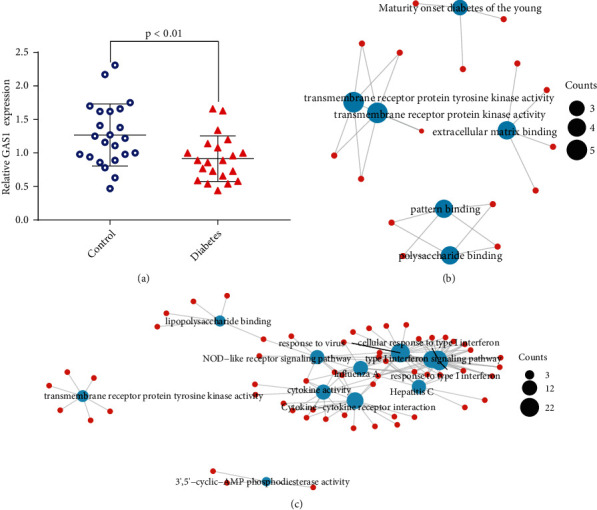
Expression and functional characterisation of GAS5 in diabetes mellitus. (a) Expression characteristics of long noncoding RNA (lncRNA) GAS5 in the serum of patients with diabetes. (b) Gene Ontology (GO) and Kyoto Encyclopedia of Genes and Genomes (KEGG) enrichment profiles of genes that were downregulated in women with GDM. (c) GO and KEGG enrichment profiles of genes that were upregulated in patients with diabetes.

The characteristics of GAS5 expression in the serum of 23 healthy and 21 patients with GDM are shown in Figure 1(a); GAS5 was downregulated in patients with GDM. Findings from the LncSEA dataset showed that the sites of subcellular localization of GAS5 were the nucleus, cytoplasm, and ribosome (Table 1). In addition, the lncRNA GAS5 was present in exosomes (Table 1). To analyse the effect of GDM on the amniotic fluid cells of the patients, we used 583 differentially expressed genes from the GSE150621 dataset for enrichment analysis. The results of enrichment analysis revealed that patients with GDM showed downregulation of genes associated with transmembrane receptor protein kinase activity, extracellular matrix binding, pattern binding, and polysaccharide binding (Figure 1(b)). Furthermore, enrichment analysis showed upregulation of lipopolysaccharide binding, viral response, cellular response to type I interferon, NOD-like receptor signalling pathway, type I interferon signalling pathway, influenza A, response to type I interferon, cytokine activity, hepatitis C, transmembrane receptor protein tyrosine kinase activity, cytokine-cytokine receptor interaction, and 3′,5′-cyclic-AMP phosphodiesterase activity (Figure 1(c)). Therefore, our results revealed that intercellular interactions, including neuropeptide receptors, are enhanced in the amniotic fluid cells of patients with GDM.

### 3.2. Common DEGs between Neuropeptide-Related Genes and GAS5 Target Protein Genes in the Amniotic Fluid Cells of Patients with GDM

**Figure 2:**
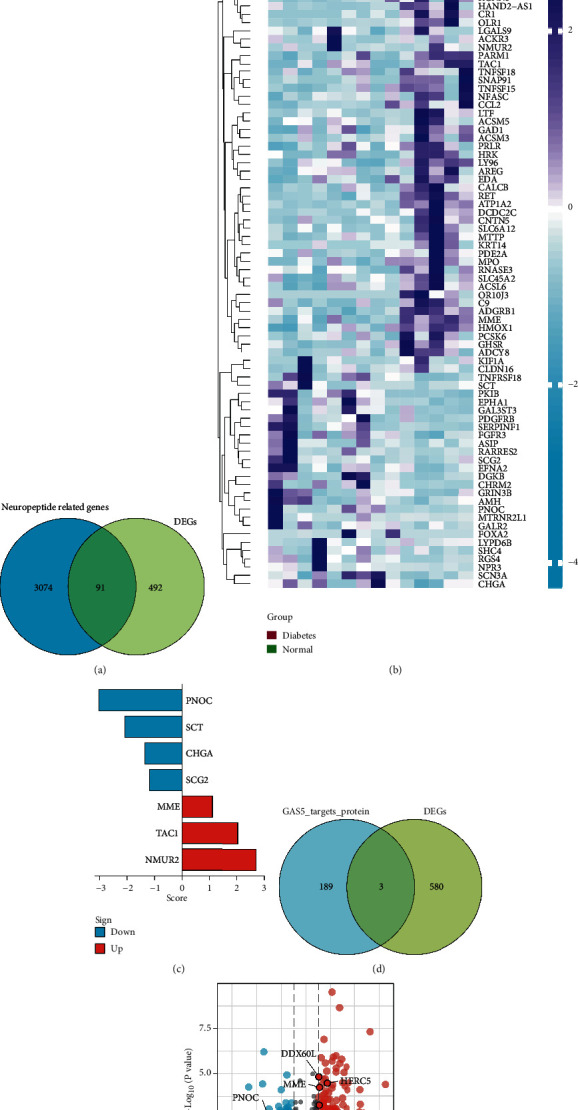
Common DEGs between neuropeptide-related genes and GAS5 target protein genes in the amniotic fluid cells of patients with gestational diabetes mellitus (GDM). (a) Venn diagram showing 91 intersecting genes among the differentially expressed genes and neuropeptide-related genes in the amniotic fluid cells of women with GDM. (b) Heat map and expression characterisation of the 91 intersecting genes. (c) Seven proteins with neuropeptide-related scores > 5 (prepronociceptin (PNOC), secretin (SCT), chromogranin A (CHGA), secretogranin II (SCG2), membrane metalloendopeptidase (MME), tachykinin 1 (TAC1), and neuromedin U receptor 2 (NMUR2)). (d) Intersection analysis of GAS5 target proteins with 91 intersecting genes was performed to identify three target proteins (DEAD box polypeptide 60-like (DDX60L), HECT and RLD domain-containing E3 ubiquitin protein ligase 5 (HERC5), and interferon induced with helicase C domain 1 (IFIH1)). (e) mRNA expression corresponding to the seven neuropeptide-related proteins and three GAS5 target proteins was plotted on a volcano map.

The Venn diagram showed that of the 583 differentially expressed genes in the amniotic fluid cells of GDM patients, 91 intersected with 3165 neuropeptide-related genes (Figure 2(a)). The expression characteristics of these 91 intersecting genes were shown in a heat map (Figure 2(b)). The seven neuropeptide-related proteins with scores > 5 included prepronociceptin (PNOC), secretin, (SCT), chromogranin A (CHGA), secretogranin II (SCG2), membrane metalloendopeptidase (MME), tachykinin 1 (TAC1), and neuromedin U receptor 2 (NMUR2) (Figure 2(c)). Intersection analysis of GAS5 target proteins with 91 intersecting genes (Figure 2(b)) was used to identify three GAS5 target proteins (DEAD box polypeptide 60-like (DDX60L), HECT and RLD domain-containing E3 ubiquitin protein ligase 5 (HERC5), and interferon induced with helicase C domain 1 (IFIH1)) (Figure 2(d)). The predicted results of the three GAS5 target proteins are shown in Table 2. The corresponding mRNA expression patterns of these seven neuropeptide-related proteins and three GAS5 target proteins were plotted in a volcano plot (Figure 2(e)).

### 3.3. Correlation Analysis, Screening, and Structure Prediction of Key Proteins

**Figure 3:**
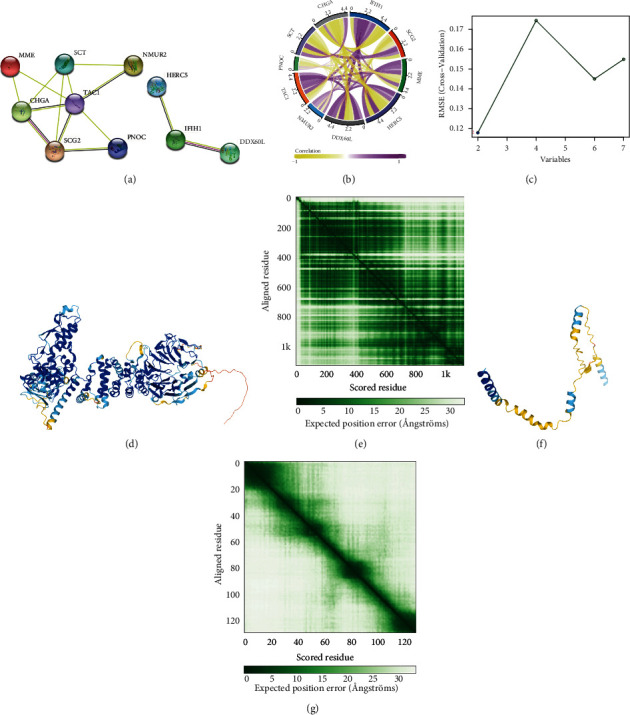
Correlation analysis, screening, and structure prediction of key proteins. (a) Key neuropeptide-related proteins and potential GAS5 target proteins were extracted. (b) Chord diagrams demonstrated the relevance of these key genes in the amniotic cells. (c) Multiple iterative support vector machines (SVM) suggested the best prediction performance was achieved when single feature factors were taken for two feature factors (HECT and RLD domain-containing E3 ubiquitin protein ligase 5 (HERC5) and tachykinin 1 (TAC1)). (d) Protein structure of HERC5 predicted using AlphaFold 2 and (e) heat map analysis of reaction prediction accuracy. (f) Protein structure of TAC1 predicted using AlphaFold 2 and (g) heat map analysis of reaction prediction accuracy.

Key neuropeptide-related proteins and potential GAS5 target proteins were extracted, and protein interaction networks were mapped (Figure 3(a)). The chord diagram demonstrated the correlation of these key genes in the amniotic fluid cells, and a positive correlation was observed between HERC5 and TAC1 expression (Figure 3(b)). Multiple iterative SVM analysis based on the GSE150621 dataset suggested that the best prediction performance was achieved when 2 feature factors (HERC5 and TAC1) were taken for a single feature factor (Figure 3(c)). Therefore, we speculated that the protein HERC5 and TAC1 may have structural interactions. Furthermore, we used AlphaFold 2 to predict the protein structures of HERC5 and TAC1 (Figures 3(d)–3(g)).

### 3.4. Prediction of GAS5/HERC5 and HERC5/TAC1 Interactions

**Figure 4:**
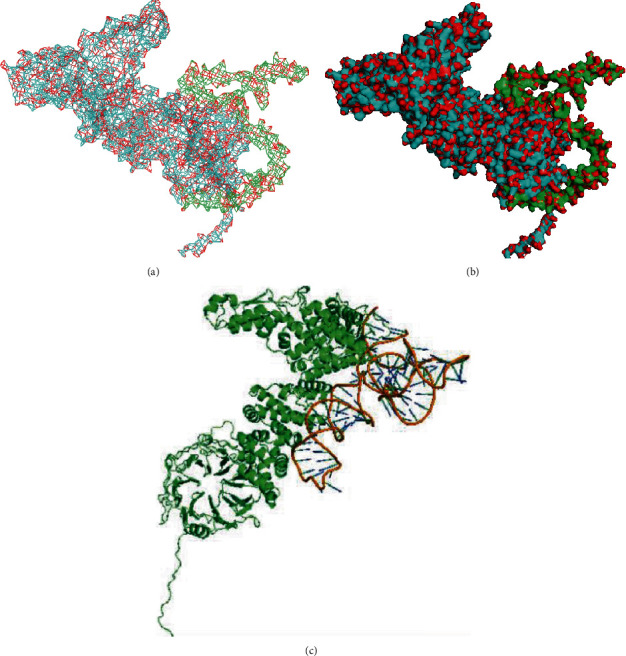
Prediction of GAS5/HERC5/TAC1 interaction. (a) Predicted HERC5 and TAC1 interaction. (b) Predicted pattern diagram of HECT and RLD domain-containing E3 ubiquitin protein ligase 5 (HERC5) and tachykinin 1 (TAC1) interaction. (c) Predicted pattern diagram of HERC5/lncRNA GAS5 interaction.

HERC5/TAC1 interactions were predicted using the ClusPro tool, and the results were visualised using PyMOL (Figures 4(a) and 4(b)). To the best of our knowledge, the HERC5/TAC1 interaction has been identified for the first time in this study. ZDOCK was used to further confirm the HERC5-GAS5 docking (Figure 4(c)). The docking score obtained using ZDOCK was 1455.958, and a docking score of ≥1000 was considered acceptable. These results indicated that GAS5 had a good docking capability with HERC5. Therefore, we proposed potential GAS5/HERC5/TAC1 interactions.

## 4. Discussion

This study assessed the potential functions of GAS5 in GDM through clinical studies and bioinformatic analyses. Furthermore, we proposed the GAS5/HERC5/TAC1 interactions for the first time in this study.

The lncRNA GAS5 expression was downregulated in patients with GDM. Intercellular interactions, including neuropeptide receptors, were enhanced in amniotic fluid cells of GDM patients. The corresponding mRNA expression patterns of seven neuropeptide-related proteins (PNOC, SCT, CHGA, SCG2, MME, TAC1, and NMUR2) and three GAS5 target proteins (DDX60L, HERC5, and IFIH1) were mapped using a protein interaction network. The multiple iterative SVM analyses based on the GSE150621 dataset suggested that the best prediction performance was achieved when the single feature factors were taken as two (HERC5 and TAC1), and the expressions of HERC5 and TAC1 were positively correlated. Therefore, we speculated that the proteins HERC5 and TAC1 may have structural interactions. Further, the HERC5/TAC1 interactions were analysed using predictions from the ClusPro tool, and ZDOCK was used to predict HERC5-GAS5 docking. Therefore, potential GAS5/HERC5/TAC1 interactions were proposed for the first time in this study.

The lncRNA GAS5 is located on chromosome 1 at 1q25 and is named after its ability to regulate key functions such as cell growth, proliferation, and survival [44–46]. Yan et al. found that the expression of GAS5 was significantly decreased in T2DM. In addition, compared with normal adipose tissue, adipose tissue of T2DM patients showed downregulation in GAS5 expression, which may explain the possible involvement of GAS5 in the pathogenesis of T2DM by regulating the transcription of IR [47]. Similarly, lncRNA GAS5 expression was significantly lower in the peripheral blood of patients with polycystic ovarian syndrome with IR and was negatively correlated with the IR resistance index of the patients [48]. Additionally, IR is a feature of GDM [49]. GAS5 expression was observed to be considerably downregulated in individuals with GDM in the clinical samples used in this investigation. Enrichment study indicates that GDM patients' amniotic fluid cells have increased neuropeptide-mediated intercellular connections. Therefore, lncRNA GAS5 was thought to be involved in the development of GDM. Venn diagram analysis indicated that GDM was related with the GAS5 target proteins (DDX60L, HERC5, and IFIH1). Additionally, we mapped PPI networks to denote the interactions between seven critical neuropeptide-related proteins and three GAS5 target proteins. HERC5 and TAC1 expression was positively linked with one another.

As an E3 ubiquitin ligase, HERC5 has a classical ubiquitin ligase activity [50]. HERC5 plays an important role in the antiviral response. *HERC5* gene expression is regulated by a variety of stimuli, particularly inflammatory factors (e.g., interferon, lipopolysaccharide (LPS), tumor necrosis factor *α* (TNF*α*), and interleukin 1*β* (IL-1*β)*) [51]. Several previous studies have shown that the incidence of GDM in pregnant women with chronic hepatitis B virus (HBV) infection is higher than that in the general population [52, 53]. HBV infection may have an impact on glucose homeostasis and IR [54]. In addition, multiple inflammatory factors are involved in the development and progression of GDM [55]. Therefore, we hypothesised that HERC5 may be involved in the pathogenesis of GDM. However, the relevance of GAS5 and HERC5 in GDM has not been clarified thus far. To the best of our knowledge, we reported for the first time that GAS5 docked well with HERC5 and both were differentially expressed in GDM. Based on the correlation between GAS5 and HERC5 with T2DM or and risk factors for T2M reported previously, we speculated that the potential GAS5/HERC5 interaction played an important role in the development of GDM.

TAC1 is an active peptide that belongs to the tachykinin class and whose gene can be selectively sheared to encode substance P (SP), neurokinin A (NKA), NKB, and neuropeptide gamma (NP*γ*) [56–59]. SP is widely distributed in the central and peripheral nervous systems of mammals. In the peripheral nervous system, SP is mainly expressed on sensory neurons in response to an injury or inflammation and is involved in the transmission of information from the peripheral injurious stimuli to the dorsal horn of the spinal cord [60]. Previous studies have reported that pain in several parts of the body is closely related to the neuropeptide SP [61–63]. Additionally, SP plays a role in the dilation of blood vessels and lowering of blood pressure [64]. NKA is a transmitter of the nonadrenergic noncholinergic (NANC) excitatory sensory neuropeptide, which is widely distributed in the central and peripheral nervous system and is involved in strong and rapid contraction of the tracheal smooth muscles [65]. In addition, TAC1 plays an important role in T2DM. Grover et al. [66] showed that serum level of SP was significantly lower in patients with T2DM than in healthy subjects. Similarly, Fu et al. found that high levels of SP were significantly associated with risk factors for obesity and T2DM [67]. However, SP has been shown to play a role in preventing complications in T2DM by reducing IR in target tissues through immunomodulation [68]. Furthermore, SP can act on inflammatory cells, leading to the release of inflammatory mediators [69]. Inflammation is an important pathological mechanism in GDM [70]. To date, however, sufficient studies to confirm the relationship between TAC1 and GDM have not been performed. In this study, TAC1 was identified for the first time as a protein with a neuropeptide-related score > 5 in the amniotic fluid cells of women with GDM and was positively correlated with HERC5 expression. Multiple iterative SVM suggested that the best prediction performance was achieved when two feature factors (HERC5 and TAC1) were taken instead of a single feature factor. Therefore, a structural interaction was thought to exist between HERC5 and TAC1. Previous studies have shown that TAC1 and HERC5 had similar functions and were directly or indirectly associated with IR and inflammatory factors, which were involved in the development of T2DM. Therefore, we hypothesised that the interaction between TAC1 and HERC5 was involved in the pathogenesis of GDM. Similarly, various studies have directly or indirectly shown that GAS5, HERC5, and TAC1 were related to IR. After calculating the protein structures of HERC5 and TAC1 using AlphaFold 2, we discovered that the ZDOCK docking score for HERC5 and TAC1 was 1455.958, indicating that GAS5 had a higher affinity for docking with HERC5 and TAC1 had structural interactions with HERC5. Therefore, GAS5/HERC5/TAC1 interactions may play an important role in the development of GDM. However, the specific mechanism underlying the development of GDM remains to be investigated.

Our results revealed that the lncRNA GAS5 was downregulated in the peripheral blood exosomes of women with GDM. The GEO database showed interactions between GAS5 and its key target proteins and neuropeptide-related proteins in the amniotic fluid cells of GDM patients. The potential GAS5/HERC5/TAC1 interaction played an important role in the pathogenesis of GDM. The limitations of this study include the following. (i) The role of the key proteins in GDM was mainly inferred by bioinformatic methods, and further studies are required to investigate their mechanism of action. (ii) Clinical samples were obtained from a single centre and the sample size was small; therefore, additional multicentre studies with a larger sample size should be performed in the future.

## 5. Conclusion

The dysregulation in the expression of lncRNA GAS5 in the peripheral blood exosomes in women with GDM may be related to HERC5/TAC1 interaction. The GAS5/HERC5/TAC1 interaction may be involved in the development of GDM, which provides important insights into the pathogenesis of GDM.

## Figures and Tables

**Table 1:** Subcellular distribution characteristics of lncRNA GAS5.

Subcellular location	Nucleus, cytoplasm, ribosome
Expression in exosomes	Positive
EVLncRNAs	Type_2_diabetes_mellitus

**Table 2:** lncRNA binding prediction to HERC5, IFIH1, and DDX60L proteins.

Gene	UniProt accession	Transcript symbol	Prediction score	Prediction *z*-score
*HERC5*	Q9UII4	GAS5-209	17.22	0.35
*IFIH1*	Q9BYX4	GAS5-209	15.84	0.13
*DDX60L*	Q5H9U9	GAS5-222	13.53	-0.24

## Data Availability

GSE150621, a dataset targeting specific RNA sequencing in amniotic fluid cells in the GDM and normal groups, was downloaded for analysis. Clinical data are available by contacting the corresponding author.
